# Voluntary information and price sharing database – a scoping review of the pricing and procurement landscape across eight Commonwealth Member States

**DOI:** 10.1080/20523211.2025.2523937

**Published:** 2025-07-15

**Authors:** Bridget Kebirungi, Phyllis Ocran, Nduta Kamere, Lynette Keneilwe Mabote, Ngozi Osi, Meghna Joshi-von Heyden, Emily Gilmour, Janneth Mghamba, Layne Robinson, Zaheer-Ud-Din Babar, Helena Rosado, Victoria Rutter

**Affiliations:** a Commonwealth Pharmacists Association, London, UK; b Phyllis Ocran, Sekondi Takoradi, Ghana; c Commonwealth Youth Health Network, London, UK; d Southern African Programme on Access to Medicines and Diagnostics, Johannesburg, South Africa; e Global Health Consultant, Meyos Health GmbH, Oberwil, Switzerland; f Commonwealth Secretariat, Marlborough House, London, UK; g College of Pharmacy, Qatar University, Doha, Qatar

**Keywords:** Medicine price, procurement, pharmaceutical supply chain, health information technology, medicine access, VIPSD

## Abstract

**Background:**

The Commonwealth Health Ministers’ meetings (2021–2023) identified medicines shortages, pricing, and price transparency as critical issues. This led to the establishment of the Voluntary Information and Price Sharing Database (VIPSD) in 2021, aimed at enhancing procurement transparency, reducing costs, and sharing information on health products amongst member states. However, adoption has been limited due to competing initiatives like pooled procurement systems introduced during the pandemic.

**Methods:**

This scoping review focused on eight Commonwealth countries: Bangladesh, Dominica, Kenya, Malaysia, Malta, Solomon Islands, South Africa, and St. Vincent and the Grenadines. Nations were selected to represent diverse health systems, population sizes, and procurement practices. External researchers conducted literature reviews and standardised data collection to ensure consistency.

**Results:**

The review revealed varied pricing policies and procurement frameworks. Nations like Malta, Bangladesh, Malaysia, and South Africa had pharmaceutical policies ensuring affordability, while others lacked comprehensive pricing policies. Seven countries implemented pooled procurement programmes, enhancing value and reducing costs; three nations operated national health insurance schemes affecting medicine pricing. Five countries used pharmaceutical management information systems to streamline procurement. Despite these efforts, disparities persist due to fragmented frameworks, stock-outs, budget constraints, and delayed procurement processes.

**Conclusion:**

The VIPSD was identified as a potentially transformative tool to enhance transparency, promote fair pricing, improve collaboration, and ensure equitable access to innovative health products. To maximise its impact, the database requires clear scope definition, comprehensive data collection, funding, and robust maintenance. Expanding its adoption could help mitigate price disparities and strengthen medicine accessibility across Commonwealth member states.

## Background

Pharmaceutical expenditures have grown exponentially and exceeded economic growth and health sector growth in many countries (Babar, 2015). The high prices of pharmaceutical items have hampered numerous health systems’ ability to deliver access to all. This leads to unavailability or disruptions in the supply of specific health products, affecting health services and ultimately resulting in adverse health outcomes for patients (World Health Organization, 2020). To guarantee access to health products, pharmaceutical financing for all sectors of the population is critical.

The lack of access to necessary medications for an estimated two billion individuals prevents them from enjoying the beneficial effects of contemporary scientific and medical advancements (World Health Organization, 2017). Lack of access to medications results in a never-ending circle of agony. The limited availability of medicine is hinged on issues relating to procurement and pricing. Additionally, there is a greater pressing need for essential medications following the global COVID-19 outbreak. In low-resource settings, patients are faced with challenges when accessing medicines. This includes systemic pharmaceutical supply chain weaknesses in procurement and distribution of medicines, contributing to suboptimal inventory management and underutilisation of these medicines (World Health Organization, 2018).

A comprehensive strategy to strengthen structures, systems, and processes in good pharmaceutical practice, logistics information management system, forecasting, quantification, procurement, warehousing, and inventory management should be developed and implemented by the pharmaceutical sector to ensure increased access. Several sides of the medicine supply chain including access, affordability, and quality of medicines can be enhanced through health systems strengthening in low and middle-income countries (LMICs) (Babar, 2021). Pharmaceutical policies promote equity and sustainability of the pharmaceutical sector by ensuring that safe, effective, and good quality medicines are accessible, affordable, and used rationally. One of the key challenges in the pharmaceutical supply chain is the absence of quality data that can be used to accurately forecast health product requirements, procure according to plan and monitor in-country stocks to avert shortages. Price transparency assists policymakers and researchers with the provision of reliable price information, enables improvement in economic efficiency, allows buyers to have strategic negotiations, and ensures price accountability with pharmaceutical firms (Kemp & Schondelmeyer, 2000). Publicly accessible data on health product pricing that can form the basis of policies and strategies in countries is limited. To establish transparent pricing for health products, it is essential to generate and supply strong, high-quality data on the pricing interventions, affordability, and accessibility of health products. This data will aid decision-makers in determining a very effective pricing model for health products.

It is imperative to develop systems that will provide data to promote transparency and accountability.

The Voluntary Information and Price Sharing Database (VIPSD) is an electronic database, launched in 2021, to support the procurement of health products at fair prices and assured quality, with an emphasis on price transparency, and sharing of information on products, suppliers, and supply chains amongst Commonwealth member states. The initiative followed the 2018 Commonwealth Health Ministers request to establish an information-sharing mechanism between member states, with the development initiated by the Commonwealth Secretariat. The VIPSD aims to increase equitable access to quality health products using transparent procurement data to inform decision-making when sourcing. The creation and implementation of the VIPSD has been endorsed by successive Ministries of Health, during the annual Commonwealth Health Ministers’ Meetings in 2021 and 2022.

To date, there has been limited deployment of the database in member states, in part due to competing initiatives and changing dynamics in the procurement landscape such as pooled procurement initiatives that were set up during and post-COVID-19 pandemic. In 2023, the Commonwealth Health Ministers meeting highlighted several issues around medicines shortages, of which pricing and price transparency were cited as some contributory factors. The VIPSD was seen as a valuable resource to help address some of these issues.

Following this call, the Commonwealth secretariat commissioned a review of the procurement and pricing landscape in selected member states to understand how the VIPSD can support procurement systems in member states amidst the plethora of existing Information Technology (IT) systems and procurement databases of the Commonwealth supply chain systems. In parallel, the Commonwealth Pharmacists Association (CPA), an accredited organisation of the Commonwealth was appointed to undertake the scoping review and help establish the Commonwealth-wide Heads of Procurement (HoP) Network to provide the required expertise, using country-specific experiences, and to further develop and deploy the VIPSD.

This scoping review contributes to academic knowledge and the findings can support quality pricing data, and innovative interventions by governments and stakeholders to improve affordable access to health products.

### Aim of the scoping review

The aim of this scoping review was to explore the procurement landscape across the Commonwealth and establish how the VIPSD could support procurement systems in selected member states.

### Objectives of the scoping review

1. To provide an overview of the pricing landscape for health products.
2. To identify the procurement landscape, including the gaps and challenges experienced by member states in procuring health products.
3. To explore the scope and functionality of current IT systems and databases used in member states for the procurement and pricing of health products.
4. To determine the use of the VIPSD in the procurement of health products.

## Methods

This scoping review aimed to explore the procurement landscape of medical products and establish how the VIPSD can support procurement systems in select Commonwealth member states. This review was not intended to be exhaustive but rather focused on mapping key themes across eight countries. Due to the limited availability of peer-reviewed literature in this area, we drew on a wide range of trusted sources – including government publications, reports from Ministries of Health, and other grey literature – to ensure a meaningful and contextually grounded synthesis.

### Country selection

Eight Commonwealth member states, out of a total of 56, were selected from the Commonwealth regions of Africa, Asia, Europe, the Pacific, the Caribbean, and the Americas for this scoping review: Bangladesh, Dominica, Kenya, Malaysia, Malta, Solomon Islands, South Africa, St. Vincent and the Grenadines. The countries were selected to represent health systems’ diversity, representative of larger and smaller populations, Commonwealth membership, as well as established and developing procurement pathways. These countries were selected for inclusion based on a combination of factors including availability of published data, healthcare system diversity, geographic representation, and relevance to pricing and procurement processes. Efforts were made to ensure representation across different income levels (e.g. high- vs. middle/low-income countries) and healthcare infrastructure types (e.g. publicly funded vs. private-dominated systems). Additionally, countries with Commonwealth membership were prioritised. This strategic selection aimed to capture a wide spectrum of VIPSD applications across varied healthcare delivery contexts.

### Data collection

External researchers, with expertise in procurement, were commissioned to conduct the desktop literature review across each country and to complete a standardised proforma, ensuring consistency in the data review. The scoping proforma was structured in five sections. Section 1: Stakeholder mapping – Key country information. Section 2: National procurement and pricing processes. Section 3: Current procurement and pricing database in-country, Section 4: Scope of current databases. Section 5: Wider Issues to consider in this scoping exercise. A systematic search was conducted across electronic databases to identify original research articles published in English on the procurement and pricing landscape of the eight Commonwealth countries. The key search words were pharmaceuticals, vaccines, procurement, pricing, information technology, database and registration, regulation, and substandard medicines. References of retrieved articles were assessed for further relevant evidence that may have been missed in the searches.

The inclusion criteria were: stakeholders in the procurement and pricing of pharmaceuticals in the public sector; the procurement and pricing of pharmaceuticals, vaccines, and other goods; IT systems and databases used for procurement and pricing; the scope of the national database(s) used for procurement; issues to consider when undertaking in-country scoping such as medicines registration, regulation and detection of substandard medicines, etc. The exclusion criteria were: magazines, editorial letters, lectures, and other publications that did not provide the relevant data or any of the outcomes listed as part of the inclusion criteria were excluded, as well as those articles not available as full text. The initial results were collated onto a spreadsheet and screened for eligible studies. All selected articles in the first stage were read to determine their relevance. Duplicate articles were removed. The results were then peer-reviewed for errors in spelling and line combinations. All articles considered potentially eligible were read in full to determine their relevance according to the inclusion criteria. At the full-text review stage, studies not meeting the inclusion criteria were excluded. All included studies were listed in the review, along with descriptions of their key characteristics.

### Data analysis

We reviewed the publications to ensure that a narrative synthesis produced was sourced from the most complete collection of relevant publications possible. Thematic analysis of the articles was conducted, and relevant sub-categories were created for examination until no more themes were identified and saturation was deemed to be reached.

## Results

The literature review findings are presented in the form of **three overarching themes**:

1. Pricing landscape of pharmaceutical medicines, vaccines, and other products;
2. Procurement landscape of pharmaceutical medicines, vaccines, and other products;
3. Scope, and functionality of available IT systems and databases for pricing and procurement.

### Pricing landscape of pharmaceutical medicines, vaccines, and other products

The results of the pricing landscapes in the eight countries chosen for the scoping review showed a diverse range of pricing policies, challenges, and approaches to ensure affordable access to medicines. Three countries (Malaysia, Malta and South Africa) had national pricing policies, three countries (Bangladesh, Dominica and Kenya) did not have any national pricing policy and for two countries (Solomon Island, and St Vincent and Grenadines) the information available was limited. While some countries had well-defined policies and mechanisms, others lacked comprehensive frameworks, leading to disparities in medicine prices. Across all countries, the review highlighted the complexities and variations in pharmaceutical pricing strategies and how they can subsequently affect the procurement and supply chain.

Bangladesh lacked a comprehensive pricing policy and was found that there was limited government control over medicines’ pricing outside of the essential medicines on the primary health care list. The price fixation committee of the Directorate General of Drug Administration (DGDA) set the maximum retail price for 117 essential medicines on the primary health care list of 1993, based on the cost of the raw material and packaging plus a mark-up of 150% to 340%, depending on the formulation. The pharmaceutical industry sets the price for all other medicines, creating a complex pricing landscape in the pharmaceutical sector. A comprehensive study analysing public healthcare facilities, private retail pharmacies, and private clinics in six regions in Bangladesh showed that the majority of medicine prices in the private sector conformed with international standards and were not excessively priced. However, some medicines aligned with international benchmark references and consistently maintained elevated price levels in both public and private sectors (Kasonde et al., 2019). Price disparities make it challenging to ensure fair access to medicines for consumers beyond those on the essential list. This is particularly evident in the pricing of medicines that consistently exceed international reference prices, which significantly impacts healthcare affordability for the population.

In **Dominica**, the burden of purchasing life-saving medicines falls heavily on individuals due to the absence of a formal national pricing policy and legal or regulatory provisions affecting the pricing of medicines. The government does not run an active national medicines price monitoring system for retail prices. Regulations do not exist mandating that retail medicine price information should be publicly accessible (Thomas, 2012). The price of pharmaceuticals can be freely determined, with the government’s Drug Regulatory Authority (DRA) oversight, often with vast differences between the prices charged by private pharmacies and international reference prices (Pan American Health Organization, 2007). With the implementation of the unofficial medicines policy, all patients receive medications at no cost at government health care facilities. The government has implemented the National Health Insurance Scheme (NHIS) to provide at least partial coverage for medicines on the Essential Medicines List (EML) (Rathe, 2010). Private health insurance schemes provide different levels of medicine coverage. The NHIS and contributive health insurance cover both the public and private sectors (Pimenta & Rezai, 2016). The lack of a national medicines price monitoring framework and trust in public services further complicates pricing challenges.

**Kenya** revealed a different approach, abolishing pharmaceutical price controls in 1994, and allowing a free pricing environment in the private sector. The pricing of medicines in the private, profit-driven healthcare sector was determined by an unregulated market approach, which employed a societal price increase that lacked formal legislative backing. There was a high mark-up of medicines in the private sector. The public sector procurement of pharmaceutical products was governed by the Public Procurement and Asset Disposal Act (2015), revised in 2022, based on the principle of the lowest bidding price (Kenya Public Procurement Regulatory Authority, 2015). Prices were often more restrained in the public and non-profit sectors, with numerous regulatory frameworks such as the Public Procurement and Asset Disposal Act. There were no import tariffs on both locally manufactured and imported pharmaceuticals. The government also announced the exemption of various medical equipment and apparatus from value-added tax (VAT), in its 2022/2023 budget, which continued to drive and encourage investment in the health sector in Kenya (Deloitte, 2023). These had significantly improved pricing in the public and non-profit health sectors.

In **Malaysia,** the prices of medicines in the public sector were indirectly controlled through government procurement guidelines, consumer price guides, and the medicines price mechanism. The pricing of medicines in the private sector depended entirely on the market forces. Each distribution level was free to set its selling price. The pricing mechanism involved setting maximum prices at two levels of the pharmaceutical supply chain, namely wholesale and consumer prices. The determination of maximum wholesale prices would consider the factory price, External Reference Pricing (ERP), and price negotiations. A regressive mark-up tier would be applied to the maximum wholesale price to determine the maximum consumer price. The maximum wholesale and consumer prices would be gazetted and made available in the public domain for reference. The respective dispensing outlets would also be required to display their selling prices publicly. The wholesalers and retailers were still free to set prices up to the capped level. This pricing mechanism was to create competition, and consumers may take advantage of the best prices in the market. Notably, the mechanism promoted transparency in medicines prices. It also provided informed choices to consumers in obtaining medicines at the best prices. Despite these mechanisms, the country faced high medicine prices within the supply chain in the absence of government interventions (Hassali et al., 2012). High medicine prices were markedly obvious in the private sector (Babar et al., 2007).

**Malta** presented a model where a comprehensive pharmaceutical policy, Medicine Act III of 2003, and references such as Maximum Reference Price (MRP), ERP, and Guidance Reference Price (GRP) were established, aimed at promoting equity and sustainability of the pharmaceutical sector and ensuring that safe, effective, and good quality medicines were accessible, affordable, and used rationally (Government of Malta, 2003; 2021b). The Government Health Services (GHS) in Malta, funded through taxation, provided free service to entitled patients, and it was 100% reimbursable (i.e. there is no co-payment). Non-entitled patients purchased pharmaceuticals fully out-of-pocket through privately owned pharmacies. Pricing computations were performed within the Directorate for Pharmaceutical Affairs (DPA) to serve as a reference price control during the procurement process of all medicines. Research of medicine prices was conducted from a basket of European Union (EU) countries e.g. North Macedonia, Malta, Slovenia, and Greece falling within the bracket of ±20 percentage points of Malta’s Gross Domestic Product (GDP) per capita in Purchasing Power Standards (PPS) using EUROSTAT figures, together with the UK price. Pricing computations also served as guidance for Health Technology Assessment (HTA) reviews of new medicines proposed for introduction onto the Government Formulary List (GFL) (Government of Malta, 2021a). Private medical practices, hospitals and pharmacies set their pricing, and these could vary significantly.

For **The Solomon Islands,** specific information on the availability of a medicines pricing policy could not be obtained**.** However, the government, through public funds, has ensured free access to medicines and medical supplies within all public health facilities.

**South Africa** was found to have a well-established medicines pricing policy. Various mechanisms – such as the Single Exit Price (SEP), External Price Referencing, selection of reference countries price adjustments, a tender system for the public sector procurements, transparency and negotiations and generics promotion – have been implemented to ensure the affordability and accessibility of medicines and vaccines. The pricing committee for the Department of Health (DoH) in South Africa played a key role in determining the maximum prices that could be charged for essential medicines. The Medicines and Related Substances Act (Republic of South Africa Government, 2017); has been central to the regulation of medicines in the country. As part of this Act, regulations have been established to bring transparency to the pricing of medicines and related scheduled substances. One of the key components of this transparent pricing system was the Single Exit Price mechanism. The SEP represented the price at which manufacturers and importers could sell medicines to wholesalers or retailers (Gray & Suleman, 2015; Moodley & Suleman, 2019). The public sector procured a significant portion of medicines and vaccines, through a centralised tender system. This bulk procurement system allowed for volume purchases at lower prices, leveraging economies of scale. These systems, alongside the tender system for the public sector, have been effective, South Africa still faced challenges, such as regulatory delays and supply chain inefficiencies, highlighting the complexities of pricing even within a structured system (Ngozwana, 2016).

**St. Vincent and the Grenadines** lack a formal national medicine pricing policy. However, there were legal or regulatory provisions affecting the pricing of medicines. These provisions were aimed at the level of wholesalers and retailers. Medicines were price controlled in the private sector with a 12% rate at the wholesale level and a 13% rate at the retail level (Jack, 2012). The government ran an active national medicines price monitoring system for retail prices. Some regulations mandated public accessibility of retail medicine price information, and the Consumer Affairs Department periodically published the information on the radio. The Caribbean Pharmaceutical Policy (Pan American Health Organization, 2013) comprised four areas: pharmaceutical policy scope, regulatory framework, access and rational use of medicines, whereby regulatory provisions covered both the public and the private sectors.

### Procurement landscape of pharmaceutical medicines, vaccines, and other goods

This scoping review showed a diverse array of legislative frameworks, models, successes, and challenges. This section highlights specific examples to underscore countries’ unique approaches and noteworthy achievements.

In **Bangladesh**, the government managed most of the procurement activities, primarily through the Central Medical Stores Depot (CMSD) for centralised procurement, and localised procurement was done by the civil surgeon’s office and individual hospitals. The CMSD supplied medicines to district hospitals, 64 civil surgeons (in charge of health facilities below the level of district hospital), and the public health programmes operated by 9-line directors in the directorate general of health services. Tertiary hospitals were not obliged to procure from the CMSD, but they were obliged to purchase from about 40 manufacturers pre-qualified by the CMSD. Central procurement involved three main suppliers: Essential Drug Company Limited (EDCL), CMSD, and local purchases. Procurement was done annually based on quantification estimates from past supply. A national or international bidding process was done depending on the procurement threshold. Challenges included stock outs caused by budget constraints and non-adherence to World Bank’s procedures. The lack of pharmacists made it extremely difficult to monitor consumption accurately, and expiries resulting in poor quantification often led to stock-outs.

Bangladesh has taken substantial steps to improve its procurement processes, and partnered with organisations such as the United States Agency for International Development (USAID) Medicines, Technologies, and Pharmaceutical Services Programs. These collaborations led to more efficient procurement and quicker availability of medical products into public health services, due to decentralisation, streamlined approval processes, and optimised evaluation reports, resulting in more efficient procurement and quicker availability of medical products in public health services. This was a notable success story in enhancing healthcare procurement efficiency.

**Dominica** had navigated its challenges through a collaborative approach, implementing the pooled national procurement programme in 2012, which had significantly improved the coordination and financial analysis of procurement. Before 2012, the estimation of needs for procurement was conducted independently by health facilities and disease control programmes, resulting in fragmented procurement with no financial analysis.

Since 2012, the Systems for Improved Access to Pharmaceuticals and Services Program (SIAPS) has assisted in implementing a coordinated national exercise for the estimation of needs, using a standardised methodology. Public procurement is conducted on a highly decentralised basis, at the level of individual spending ministries, local authorities, or other public bodies covered by the procurement law (Dominican Republic Ministry of Public Health, 2015). However, in some cases, there is a mixed approach with elements of centralisation (Dominican Republic Ministry of Public Health, 2015). The public sector conducts the national pooled (bulk) procurement of medicines, bulk quantities of generic categories of goods, and supplies through the central medical store, Programa de Medicamentos Esenciales/Central de Apoyo Logístico (Program for Essential Medicines/Central Logistics Support – PROMESE/CAL).

Public sector procurement in Dominica is mainly conducted through the Organization of East Caribbean States/Pharmaceutical Procurement Service (OECS/PPS) and approximately 95% of pharmaceuticals are procured through it (Thomas, 2012). In 2010, the Ministry of Health endorsed an integrated, single-supply system for managing medicines and medical supplies (SUGEMI, in Spanish) (Thomas, 2012). The SUGEMI information system collects and reports information on consumption and stocks available but does not have an automated inventory management system in the warehouses and pharmacies (Thomas, 2012).

In **Kenya**, medicines procurement in the public health sub-sector is mainly done through the Kenya Medical Supplies Authority (KEMSA), responsible for purchasing around 30% of all prescription drugs in the Kenyan market and procuring medicines for some donor partners. The procurement system is decentralised, with each procuring entity conducting procurement procedures separately, using standardised tender documentation. The private sector’s procurement of medicines is not as significant as the public sector. In the private sector, medicines procurement is done by various private healthcare providers, pharmacies, and other organisations. One example is the Mission for Essential Drugs and Supplies (MEDS), a faith-based organisation that procures and supplies essential medicines and medical supplies to private healthcare facilities in Kenya. Additionally, MedSource, a private sector initiative in Kenya, negotiates prices on behalf of its members and allows them to buy at negotiated prices. Some non-governmental organisations (NGOs), such as Médecins Sans Frontières (MSF) and the Red Cross, use a multi-country pooled procurement system. On average most county health facilities procure 70% of their medicines from KEMSA, 28% from MEDS, and 2% from private for-profit distributors.

Kenya has several competitive procurement strategies, guided by numerous regulatory frameworks such as the Public Procurement and Asset Disposal Act (Kenya Public Procurement Regulatory Authority, 2015), to significantly improve pricing in the public and non-profit health sectors. Kenya has also introduced innovations such as electronic Local Purchase Order (LPO) generation and centralisation, which have enhanced procurement efficiency and reduced costs. Kenya serves as an example of how diverse procurement strategies can be effectively employed.

In **Malaysia**, the Ministry of Health Medicines Formulary (Malaysia Ministry of Health, 2015) controls and promotes the rational and quality use of medicines in public healthcare facilities. Public facilities can procure approximately 350 medicines, which are listed in the Approved Product Purchase List (APPL), directly from Pharmaniaga Logistics Sdn Bhd through the concession agreement at prices negotiated by the Ministry of Health. Some medicines procurement involves the Ministry of Health’s facilities ordering medicines via tenders managed centrally by the procurement division with technical support from the pharmaceutical services programme. Some direct local procurements can be done by facilities for low value purchases from registered suppliers. Malaysia has adopted a bulk procurement model, including a national concession agreement and centralised tenders, which has proven successful in achieving value for money in public-sector medicines procurement.

**Malta** has leveraged technological advancements in its procurement processes. Procurement of all pharmaceuticals listed in the Government Formulary List (GFL) for all hospitals is procured centrally by the Government Health Procurement Services (GHPS) via a tendering process. The widespread use of an e-procurement system for public health facilities and its regular updates have streamlined the acquisition of pharmaceuticals.

In **South Africa**, the public healthcare sector serves the needs of more than 82.5%–84% of the population (60 million); this accounts for 16% (ZAR 6.1 billion) of total pharmaceutical expenditure, and access to 2400 product lines (Modisakeng et al., 2020; Organisation for Economic Co-operation and Development, 2018). The public sector procurement of medicines and pharmaceutical supplies is undertaken through an open centralised tender system. The centralised tender system has been largely successful in standardising procurement processes and achieving economies of scale. The National Department of Health enters contracts with pharmaceutical suppliers on behalf of the provinces (Gray et al., 2017). Hospitals may sometimes resort to buy-outs to acquire medicines listed on the EML that are not part of the tender agreement. The private healthcare sector serves approximately 17.5% (8 million) through private insurance schemes, and accounts for roughly 80% of total pharmaceutical expenditure (ZAR 33.1 billion), supplying 5000 product lines (Modisakeng et al., 2020; Organisation for Economic Co-operation and Development, 2018). The private hospitals and pharmacies obtain their medicines through private distributors or directly from pharmaceutical companies. The introduction of the SEP system and centralised competitive tendering has been instrumental in ensuring a consistent supply of essential medicines.

In the **Solomon Islands**, there is a defined medicines procurement policy, using the Central Medical Store (CMS) model, for the procurement and supply of medicines, medical devices, and pharmaceuticals. The country leans towards experienced, foreign-based preferred suppliers for essential medicines and non-consumables. The Solomon Islands’ commitment to competitive tendering and adherence to principles of competition, transparency, fairness, and equal opportunity has led to a reduction in sole source contracting. This highlights the importance of adhering to ethical principles in procurement, even in resource-constrained settings. While the Ministry of Health and Medical Services strives to ensure universal access to essential medicines and supplies, they face challenges with distribution, limited funding for the enforcement agencies to effectively regulate medicines and ensure their safety, efficacy, and quality of products throughout the supply chain without hampering the procurement targets (Solomon Islands Government, 2023).

**St. Vincent and the Grenadines**, a small island state, is part of the Organization of Eastern Caribbean States, which collaborates on intergovernmental-led pooled procurement efforts to ensure the availability of essential medicines. The Central Medical Stores, which uses the Organization of Eastern Caribbean States/Pharmaceutical Procurement Service pooled procurement services, centralises the procurement of goods and services for the public sector. There are more than 93% of key medicines always available in-country at the CMS (Jack, 2012).

Despite limitations in resources, the use of such pooled procurement services has been successful in overcoming procurement and distribution challenges, as well as building stability and quality into the procurement process.

### Key procurement challenges

The eight countries analysed reflected a mix of legislative structures, procurement models and challenges across the procurement landscape. Some of the key procurement challenges identified are summarised in Table 1.

**Table 1.** An overview of key procurement challenges experienced across eight Commonwealth countries.

**Stock-Outs and Shortages**	One of the most pressing issues in many of the countries reviewed was the frequent occurrence of stock-outs and shortages of essential medicines. The unavailability of critical drugs compromises the quality of healthcare.
**Budgetary Constraints**	For most countries, limited financial resources allocated to procurement often result in insufficient funds to acquire necessary pharmaceuticals. This leads to a constant struggle to meet the demand for medicines, particularly in public healthcare systems.
**Lack of Transparency**	Transparency issues in the procurement process can foster corruption and unethical practices. When transparency is compromised, it erodes public trust and jeopardises the fair and efficient allocation of resources.
**Poor Inventory Management**	Inadequate inventory management systems can lead to inefficiencies, such as overstocking or understocking of medicines. These errors not only impact costs but also affect the availability of essential drugs.
**Delayed Procurement Processes**	Bureaucratic delays in procurement can slow down the acquisition of medicines, leading to critical delays in their delivery to healthcare facilities.
**Coordination Challenges**	Poor coordination between different levels of healthcare systems, especially in countries with decentralised procurement models, can lead to inconsistencies in the availability of medicines. Lack of coordination can also hinder the pooling of resources for bulk procurement.
**Dependence on Donor Funding**	Some countries face a heavy reliance on donor funding for medicines procurement. This dependency makes procurement vulnerable to external factors and can disrupt the consistent supply of drugs.
**Obsolete Procurement Guidelines**	Outdated procurement guidelines in some countries can fail to address the evolving needs and challenges of healthcare systems. This can hinder the adoption of modern, efficient procurement practices.
**Limited Qualified Personnel**	Shortage and high turnover of qualified pharmacists and procurement professionals often lead to inefficiencies noted in quantification, procurement decisions, and inventory management.
**Infrastructure and Distribution Challenges**	Many countries, especially those with remote or underserved areas, face difficulties in the timely distribution of medicines. Infrastructure challenges and the impact of external factors, such as the COVID-19 pandemic, can disrupt procurement and delivery.

### Scope, remit, and functionality of available IT systems and databases for pricing and procurement

Across the eight countries, there are numerous IT systems and databases currently in use, as shown in Table 2, with each country facing unique challenges in their pricing and procurement pathways (Table 3).

**Table 2.** An overview of the use and data available in various IT systems and databases for pricing and procurement of health products across eight Commonwealth countries.

Country	Available IT System/ Databases	Uses and Data captured	Access
Bangladesh	Supply Chain Management Portal (SCMP).	Comprehensive procurement, planning, package development, tracking commodities, and forecasting family planning/reproductive health commodities.	Closed.
Dominica	SUGEMI Pharmaceutical Management Information System (PMIS).	Procurement data, and pharmaceutical management activities.	Not defined.
Kenya	IFMIS, KHIS, OpenLMIS, eLMIS.	Procurement and supply chain data.	Restricted access to authorised personnel.
Malaysia	E-Procurement. Consumer Price Guide.	Procurement data showing e.g. active ingredients, dosage, pack size.	Not defined.
Malta	ePPS (Electronic Public Purchasing System).	National procurement management including drug prices.	Closed system for validated users.
South Africa	MPR (Medicine Price Registry), CSD, e-Tender.	Maximum medicine prices, supplier information, and procurement data.	CSD is closed, MPR and e-Tender are open to the public.
Solomon Islands	mSupply.	Procurement data including medicine quantities and prices, inventory management, forecasting of essential medicines, and procurement functions, including quantification, tenders, purchase orders, and goods receipt.	Not defined.
St Vincent and the Grenadines	Procurement website.	Procurement notices, awards, requests for bids and proposals, prequalified suppliers list, and products and quality testing data.	Not defined.

**Table 3.** Key challenges identified across current IT systems and databases used for pricing and procurement in eight Commonwealth countries.

**1. Data Accessibility and Integration**	Closed Systems. Fragmentation. Lack of seamless data – resulting in limited data driven decision-making.
**2. Data Quality, Completeness and Transparency**	Use of different databases for similar activities. Lack of completeness – data entry issues leading to reduced accuracy in decision making.
**3. Infrastructure and Connectivity**	Power supply and unstable internet – particularly in remote areas. This affects the usability and effectiveness of IT systems.
**4. Language and User Interface**	Poor user-friendliness of systems affects utilisation. Often complex technical language for non-technical staff.
**5. Workforce Retention and Technical Expertise**	Technical capacity to manage systems. High turnover impacts ownership.
6. **Deployment and Capacity Building**	Limited user training and capacity-building programmes – for healthcare workers and system users. The complexity of IT systems leads to inadequate deployment.

**Bangladesh** demonstrates a comprehensive approach with the Supply Chain Management Portal (SCMP) and the electronic Logistics Management Information Systems (eLMIS) which enables procurement planning of goods and services, product identification through the catalogue tracking of packages, linkage with drug registration database, and forecasting of health commodities, to ensure a stable supply (Kibria et al., 2016). The SCMP is a web-based portal accessible to the Ministry of Health and Family Welfare (MOHFW), all procuring stakeholders and donors. The Upazila Inventory Management System and the Warehouse Inventory Management System (WIMS) are also used to monitor commodity status at service delivery points (SDPs) and inventory at district-level family planning warehouses respectively.

Key challenges centre around issues such as workforce, with a lack of technical personnel and a high turnover of officials that hinders general systems management and decision making (Ahmed et al., 2015; Kibria et al., 2016; Twesigye et al., 2017).

**Dominica** uses the SUGEMI Pharmaceutical Management Information System (PMIS), which has information on prices and is available at the national, regional, and facility levels (Systems for Improved Access to Pharmaceuticals and Services, 2017) and is used for procurement and support forecasting. The system has improved the efficiency of pharmaceutical management, ultimately contributing to an increased availability of essential medicines in health facilities (Barillas, n.d.). It has also decreased the frequency of stock-outs and wastage of unused, expired commodities while lowering overall purchasing prices for these drugs and supplies. Procurements are managed through the Dirección General de Contrataciones Públicas (DGCP) portal (Martínez et al., 2021). The portal tracks information about procurement data from all the local and central government agencies buying using the Portal Transaccional and the Registro de proveedores del Estado (Dominican Republic n.d.a and n.d.b). It also contains the contracting processes of all local and central government agencies that use the transactional portal.

Key challenges in Dominica include the use of technical language when evaluating supplier proposals and the language selection criteria, which can make it difficult for those without technical expertise to understand and engage in the procurement processes. These could potentially be addressed by providing simplified definitions of the proposal and process, along with language selection options.

**Kenya** utilises multiple systems, including dedicated options for vaccines, to manage procurement, track inventory, and oversee supply chain logistics. The Integrated Financial Management Information System (IFMIS), widely across various Kenyan government departments, plays a key role in enhancing transparency and accountability in the public procurement of medicines. The Kenyan government’s Public Procurement Information Portal (PPIP) aims to improve transparency in the procurement processes. The Kenya Health Information System (KHIS), built on the District Health Information Software 2 (DHIS-2) platform, is used to collect, analyse, and disseminate health data, including information related to medicines and medical supplies at the health facility level. The Open LMIS (logistics management Information system) is used for the Health Commodity Management Platform and Centralized Harmonized Automated Networked Governance Optimization (CHANGO) is used for vaccines. The eLMIS is used for vaccine supply chain management, cold chain analytics, and decision-making in Kenya. There is no national online database for medicine pricing information. Pricing data is shared through government official channels, KEMSA and the Ministry of Health. Challenges in accessing information or user functionality in these procurement management systems include limited access, poor data quality, poor infrastructure connectivity, limited data integration, and interoperability, lack of real-time data availability, non-user-friendly interfaces, and features, lack of customisation and scalability, lack of maintenance, lack of poor data security and privacy.

**Malaysia** employs an e-procurement database (Hamzah et al., 2020) that covers the Ministry of Health’s facilities, with pricing data shared via the Consumer Price Guide (Malaysia Ministry of Health, 2022). The database records APPL purchases for the Ministry of Health’s facilities (hospitals, special medical institutions, health clinics and community clinics) provided directly by the supplier, as well as national tenders and direct purchases provided by individual facilities (Hamzah et al., 2020). Limited documentation exists on challenges related to accessing information or using the database. However, key issues include limited collaboration between relevant government agencies, healthcare providers, and pharmaceutical companies in establishing fair pricing practices and ensuring access to essential medicines.

**Malta** has a national Electronic Public Purchasing System (ePPS) (Government of Malta, n.d.a ) for national procurement which adheres to the provisions of the European Union. The ePPS is an eProcurement/ eSourcing tool used by public authorities at the national, regional, and facility levels respectively for procurement of supplies, services, and works, and covers the entire procurement cycle including information on prices (Government of Malta, n.d.aand n.d.b). Key challenges include a lack of documentation for the completion of work stages requiring physical signatures leading to potential delays and language issues (Azzopardi-Muscat et al., 2017; Organisation for Economic Co-operation and Development, n.d.)

**South Africa** has several systems including the Medicine Price Registry (MPR) (South African Department of Health, 2024) which provides open access to maximum medicine prices, enhancing transparency. The Central Supplier Database (CSD) is a closed system providing supplier information, while e-Tender (e-Tender, n.d.) provides information on government tenders. The National Surveillance Centre (NSC) dashboards are used to provide visibility of medicine availability across all levels of the public health supply chain through the implementation of the Visibility and Analytics Network model to monitor medicine availability by visualising the data that suppliers and health establishments provide in a way that can help to inform their medicine supply decision-making processes. South Africa has been working on the National Pharmaceutical Information System (NPHIS) to provide a comprehensive view of the pharmaceutical environment, and procurement data. While these systems offer valuable data, challenges like limited information coverage, lack of integration, and complex user interfaces exist.

**Solomon Islands** uses an electronic medicines inventory management platform (mSupply) (Dadari et al., 2022) to manage essential medicines, and provide procurement prices, quantities, and forecasts for essential medical supplies. The mSupply manages the inventory of medicines, medical devices, and pharmaceuticals, ensuring that they are available to all public health facilities. It enables users to perform key procurement functions such as quantification, tenders, purchase orders, and goods receipt. It provides data on procurement prices and quantities of tracer drugs, vaccines, and supplies, which can be used for forecasting purposes. The system allows for the comparison of procurement prices with international reasonable standard pricing, including the Price Information Exchange for Medical Products (PIMEDs). The mSupply Mobile, an extension of mSupply, allows users to perform basic stock management functions, such as issuing goods to facilities or patients, receiving goods from central stores, and conducting stocktakes using mobile phones. Key challenges include issues around the deployment, the need for system customisation, and workforce training.

**St. Vincent and the Grenadines** employ the procurement module of SmartStream, which integrates with the regional e-procurement website (Government of Saint Vincent and the Grenadines, n.d.) used for managing procurement functions such as bid invitations and awards. Challenges include the absence of unified information on the medicines available on the market.

Across all eight countries, a wide range of IT systems is in use, for different purposes such as medicines and vaccines, medicines, and laboratory supplies. In each country, these systems are mainly led and managed by their respective governments, through centralised teams. The scoping review showed varying challenges across IT systems and databases used for pricing and procurement, which have been collated and themed below.

## Discussion

The findings of this scoping review highlight the pricing, procurement and IT landscape of pharmaceutical medicines, vaccines and other products within eight countries representative of Commonwealth health systems’ diversity. The dynamics varied from country to country and this variation affected the challenges observed at country level.

### Pricing, procurement, and IT landscape of pharmaceutical medicines, vaccines, and other products

The pricing landscape across the eight countries demonstrates a diverse range of pricing policies, challenges, and approaches to ensure affordable access to medicines. High medicines prices can be traced to the lack of transparency and limited government interventions in the pricing of medicines. Other factors influencing inefficiencies of the pharmaceutical market are the absence of legal provisions, information asymmetry, lack of healthy competition, price undercutting, market monopolies, bonus offers, discounts, and unfair rebates resulting in wide price variations for the same medicines across different private healthcare facilities. These factors highlight the importance of enforcing medicines pricing policies to combat price disparities effectively (Babar, 2022; Babar, 2024).

It was noted that benchmarking and comparison to international standards are crucial for ensuring affordability, access to essential medicines, and addressing challenges such as transparency, inadequate government intervention, and supply chain efficiency. In Bangladesh, a specific subset of medicines consistently maintained elevated price levels across all sectors. Within the public sector, the medicine with the highest price, when benchmarked against International Reference Prices (IRP), was procured at a rate approximately 2.5 times higher (Kasonde et al., 2019). The burden of purchasing life-saving medicines falls largely on individual patients living in Dominica. There is a need for government intervention(s) and pricing transparency to alleviate the financial burden on citizens. In countries like Malaysia, where medicine prices are determined by free-market economics, without government control, mechanisms should be established to prevent excessive price increases by manufacturers. This would ensure greater access to essential medicines and improve the overall pricing landscape in each country.

Across all countries, the review highlighted the complexities and variations in pharmaceutical pricing strategies and how they subsequently affect the procurement and supply chain. In Malta, Governmental policies, and healthcare budgets are key determinants of affordable pricing of pharmaceuticals leading to accessibility and affordability among patients (Kochova et al., 2021). Medicines are less affordable for consumers when compared to other countries in the European Union (Malta Medicines Authority, 2015). Some studies have shown that regulating medicine prices can lead to cost reductions while maintaining sustainable healthcare spending. However, other studies suggest that medicine price controls could potentially hinder the introduction of new medicines into the market, particularly in low-income countries (Kanavos et al., 2017; Rida et al., 2017).

The different procurement approaches demonstrate that there is no one-size-fits-all approach to healthcare procurement and its related challenges, with many countries, particularly those with smaller populations and limited resources such as Dominica, St Vincent and the Grenadines, taking a collaborative approach. Bangladesh’s Directorate General of Health Services (DGHS) has successfully improved its procurement process through partnerships and collaborations (Twesigye et al., 2017). By decentralising procurement, aligning approval authority, and optimising evaluation report processes, approval times for each package were significantly shortened, saving up to 12 weeks as compared to previous procedures (United States Agency for International Development, 2022). Dominica has overcome some of the hurdles posed by its small size and limited financial resources.

The sharing of resources, such as a trained and expert workforce, price transparency across borders and bulk buying, have demonstrably streamlined procurement pathways and have improved efficiency. International collaborations and pooled procurement with international entities, such as the Global Fund or the Global Alliance for Vaccines and Immunization (GAVI) for certain vaccines, antiretroviral medicines, or other specialised drugs, lead to better pricing and access due to larger volume purchases. Caribbean countries, including St. Vincent and the Grenadines, confront unique problems when it comes to medicines procurement because of their very small size, including smaller and less desirable markets and smaller economies that limit the amount of accessible financial resources. The CMS, which uses the OECS/PPS pooled procurement services, can be considered the most successful since it always makes over 93% of essential medicines available (Jack, 2012).

A more robust national database to capture essential information on medicines pricing and procurement would be of benefit. In addition, strong regulatory frameworks around procurement and the use of technology have been beneficial in reducing price disparities and ensuring a consistent and fair supply of medicines. These valuable examples show how success arises from partnerships, innovation, and a commitment to improving transparency and efficiency across procurement pathways.

The challenges across IT systems and databases used for pricing and procurement show the need for strategic improvements and solutions. Logistical challenges can be addressed by strengthening data accessibility through better data quality, transparency, and completeness. Workforce capability improvements will entail an incorporation of capacity-building perspectives. The overall effectiveness and efficiency of systems like IFMIS, KHIS, eLMIS, and OpenLMIS in Kenya can be improved by developing and implementing data exchange standards and protocols to facilitate seamless data sharing between different systems. Adopting technologies and processes that enable real-time data collection, processing, and dissemination would further improve system performance. Additionally, developing mobile applications and offline capabilities for these systems will ensure usability in areas with poor connectivity. For example, Malta’s current IT system/database for pharmaceuticals faces challenges such as supply chain interruptions, tender non-performance, data management issues, infrastructure and logistics, financial constraints, regulatory delays, procurement inefficiencies, and transparency issues. The decentralisation of authority across provinces sometimes leads to disparities in availability and access to medicines nationwide. To address these challenges, the implementation of an integrated IT system for real-time tracking of stock levels, demand forecasting, and timely reordering to prevent stock-outs and providing training for procurement officers could be considered. Tackling these issues, alongside pricing transparency, could potentially lead to more effective pricing and procurement processes and ultimately a more seamless and efficient environment for managing essential healthcare resources.

The challenges identified in this scoping review, show the need to have a robust price-sharing database amongst selected Commonwealth countries. With the launch of the VIPSD, there is a need to operationalise it in the selected Commonwealth countries. The implementation of a Commonwealth price sharing database, the VIPSD, would contribute to the collective action on health priorities through price setting and price comparisons in the public sector, helping countries to negotiate better prices, to reduce the costs of essential medicines, vaccines, and health technologies. It would also enable the provision of information on the manufacturer’s net selling price for member states and provide a readily searchable database of real-time verified supply information on medicines, technologies, and supply chains to support informed decision-making for procurement processes. In addition, the VIPSD would support procurement methods with an emphasis on reducing costs and sharing information on products, suppliers, and supply chains.

### Limitations of the scoping review

The scoping review was restricted to documents published in English, thereby excluding any publications in other languages. It focused on the eight selected Commonwealth countries instead of the larger fifty-six Commonwealth countries, which curbed the capacity to draw broad and comprehensive deductions. There were no key informants or focus group discussions with professionals in the eight selected countries to augment the information obtained from the desk review.

Another significant limitation of this desktop review was the reliance on sources that, in some cases, may be outdated. While every effort was made to include the most relevant and recent articles, issues, and policies, some of the available literature was published several years ago. We acknowledge that the nature of these sources may limit the relevance of the insights drawn, especially where data or policy shifts may have occurred. Additionally, despite extensive efforts to identify relevant data, many areas of interest were underrepresented or absent in the existing literature and thus not captured in this review. This lack of comprehensive coverage may have constrained the depth of analysis and left critical gaps.

Notwithstanding these limitations, the review yields significant revelations about the pricing landscapes, diverse range of pricing policies and approaches to ensure affordable access to medicines in the eight countries chosen for the scoping review.

## Conclusion

This scoping review of the pricing and procurement landscape across eight Commonwealth Member States has revealed critical insights into the challenges, strategies, and opportunities in ensuring equitable access to essential medicines. The review emphasises the complexity of pharmaceutical pricing and procurement, with notable differences in practices, regulatory frameworks, and the use of IT systems to manage these processes. Despite the variation in approaches across countries, the need for transparency, efficiency and strategic collaboration emerge as a common challenge that needs to be addressed to improve affordability and availability of medicines. Several key findings emerged from this study, including:

**Diversity in pricing disparities:** High medicines prices, particularly in public health sectors, are a significant barrier to access. Factors such as limited government intervention, lack of transparency, market monopolies, and price undercutting contribute to inefficiencies and wide price variations.

**Procurement models:** Procurement systems exhibit considerable variation across countries from centralised systems and transparency tools to collaborative approaches, such as pooled procurement to overcome resource limitations.

**IT systems and databases:** A major barrier to effective procurement is the fragmented and inefficient use of IT systems. While some countries have adopted sophisticated systems, challenges such as poor data quality, lack of real-time updates, and limited system interoperability persist hampering decision-making and affecting the efficiency of the procurement processes.

**Regulatory and governance challenges:** Gaps in regulatory oversight, especially in resource-constrained settings, hinder the effective monitoring of medicines quality and supply chains. Strengthening regulatory frameworks is essential to ensure that medicines are not only available but also safe and effective.

Based on the findings from this scoping review of the pricing and procurement landscape across eight Commonwealth Member States, the use of the VIPSD will improve transparency, collaboration, data-driven decision-making, and fair pharmaceutical pricing. It will also address specific challenges and align with the overarching goals of these countries to ensure equitable access to essential medicines and health commodities. To get the best value from the VIPSD, it is anticipated that the established Heads of Procurement Network will spearhead the VIPSD initiative in their various countries and help to define its scope, remit, and applicability.

This research study will offer information, add to scientific literature, improve the knowledge, equity considerations, access, transparency, collaboration, data-driven decision-making and guide in formulating policies regarding pricing, and access to medicines within the selected Commonwealth countries.

It is hoped that the findings and recommendations of this study will contribute to the ongoing examination of the pharmaceutical sector in the selected Commonwealth countries to ensure fair pharmaceutical pricing and access to medicines.

### Implications and recommendations

The findings from this scoping review underscore the urgent need for more robust regulatory frameworks, enhanced transparency in pricing, and the integration of more efficient data management systems. Policymakers and practitioners must prioritise these areas to foster equitable access to medicines, especially in resource-limited settings.

**Improving regulatory capacity:** Strengthening regulatory frameworks and enforcement mechanisms is critical to ensuring the safety and quality of medicines throughout the supply chain. Establishing mechanisms for better price control, transparency and benchmarking can reduce the risk of inflated costs, particularly in public healthcare systems.

**Strengthening collaborative models:** Strong collaborations and partnerships are key in enhancing procurement efficiency, especially in smaller nations or those with limited resources. Pooled procurement models can leverage collective purchasing power and improve access to essential medicines while driving down costs through bulk buying.

**Enhancing data systems and technology:** Countries should invest in integrated and interoperable IT systems for procurement planning and supply chain management to allow for real-time data sharing, transparent pricing, and efficient inventory management. Training and capacity building for workforce development are also essential to ensure the effective use of these systems.

**Adopting best practices:** Health procurement practices should prioritise efficiency through streamlined processes, such fostering transparency in tendering processes, aligning procurement strategies with best international practices for better outcomes in terms of availability and affordability of medicines.

The findings of this scoping review highlight the need for a multi-faceted approach to addressing the challenges of pharmaceutical pricing and procurement. The implementation of the VIPSD could play a pivotal role in improving transparency, fostering collaboration, and ensuring the fair pricing of medicines across Commonwealth countries. The establishment of the VIPSD would also create opportunities for more informed decision-making, ultimately leading to better outcomes in healthcare procurement and access to essential medicines, ensuring that healthcare systems are more resilient, equitable, and sustainable.

### Future research studies

Given the identified themes and acknowledged constraints, there is a need for more comprehensive studies focusing on price sharing databases. Additionally, a detailed scrutiny of the healthcare infrastructures of the Commonwealth countries is essential to provide insight into the health system and what database will be applicable in each healthcare system.

## Data Availability

The datasets used and/or analysed during the current study are available from the corresponding author upon reasonable request.

## Authors’ information

The Commonwealth Pharmacists Association is the leading global network of pharmacists across the Commonwealth, seeking to develop, empower, and raise the profile of the pharmacy profession and support the safe and effective use of medicines for the benefit of the general public.
